# Web-Based AI-Driven Virtual Patient Simulator Versus Actor-Based Simulation for Teaching Consultation Skills: Multicenter Randomized Crossover Study

**DOI:** 10.2196/71667

**Published:** 2025-11-20

**Authors:** Edward G Tyrrell, Sardip K Sandhu, Kathryn Berry, Suzan F Ghannam, Sarah A Lewis, Daniel Crowfoot, Gurvinder Singh Sahota, Julie Carson, Emma E Wilson, Jaspal Taggar

**Affiliations:** 1 Primary Care Education Unit, School of Medicine, University of Nottingham, Room C34, Queen's Medical Centre, Nottinghamshire, NG7 2UH, United Kingdom, 44 1158231418; 2 Lincoln Medical School, University of Lincoln, Lincoln, United Kingdom

**Keywords:** communication skills, simulation, clinical education, medical education, technology enhanced learning

## Abstract

**Background:**

There is a need to increase health care professional training capacity to meet global needs by 2030. Effective communication is essential for delivering safe and effective patient care. Artificial intelligence (AI) technologies may provide a solution. However, evidence for high-fidelity virtual patient simulators using unrestricted 2-way verbal conversation for communication skills training is lacking.

**Objective:**

This study aims to compare a fully automated AI-driven voice recognition-based virtual patient simulator with traditional actor-based consultation skills simulated training in undergraduate medical students for differences in developing self-rated communication skills, student satisfaction scores, and direct cost comparison.

**Methods:**

Using an open-label randomized crossover design, a single web-based AI-driven communication skills training session (AI-CST) was compared with a single face-to-face actor-based consultation skills training session (AB-CST) in undergraduates at 2 UK medical schools. Offline total cohort recruitment was used, with an opt-out option. Pre-post intervention surveys using 10-point linear scales were used to derive outcomes. The primary outcome was the difference in self-reported attainment of communication skills between interventions. Secondary outcomes were differences in student satisfaction and the cost comparison of delivering both interventions.

**Results:**

Of 396 students, 378 (95%) completed at least 1 survey. Both modalities significantly increased self-reported communication skills attainment (AI-CST: mean difference 1.14, 95% CI 0.97‐1.32 points; AB-CST: mean difference 1.50, 95% CI 1.35‐1.66 points; both *P*<.001). Attainment increase was lower for AI-CST than AB-CST (by mean difference 0.36, 95% CI −0.60 to −0.13 points; *P*=.04). Overall satisfaction was lower for AI-CST than AB-CST (8.09 vs 9.21; mean difference −1.13, 95% CI −1.33 to −0.92 for AI-CST vs AB-CST; *P*<.001). The estimated costs of AI-CST and AB-CST were £33.48 (US $42.22) and £61.75 (US $77.87) per student, respectively.

**Conclusions:**

AI-CST and AB-CST were both effective at improving self-reported communication skills attainment, but AI-CST was slightly inferior to AB-CST. Student satisfaction was significantly greater for AB-CST. Costs of AI-CST were substantially lower than AB-CST. AI-CST may provide a cost-effective opportunity to build training capacity for health care professionals.

## Introduction

There is a global shortage of health workers. The World Health Organization estimated this shortfall at 15 million in 2020 [1]. The United Kingdom government plans to train greater numbers of health care professionals [2], including doubling the number of medical training places by 2032 [3]. This has significant capacity resource implications [4], especially within clinical environments [5], where lack of available space [6] and supervisor capacity [7] are important barriers to increasing trainee numbers.

Communication is a crucial skill for all health care professionals in order to provide safe, effective, and patient-centered care [8]. Good practitioner communication helps improve patient well-being [9], shared decision-making, treatment compliance, health-promoting behaviors [10], patient satisfaction, and reduce health care litigation [11]. Therefore, building capacity for communication skills training will be required in parallel with the increasing number of health care professionals being trained.

Complex skills, such as communication, are most effectively learned through experiential methods such as facilitated simulation through role-playing with trained patient actors [12] or live clinical patient encounters with feedback and debriefing [13]. However, these approaches are resource-intensive on physical space, staffing, materials, organization, and timetabling [14,15]. In addition, differences in actor performance or patient presentation manifest as variability of student experiences and assessment standardization [16].

Novel technologies such as virtual patient simulators (VPSs, defined as interactive computer simulations of real-life clinical scenarios for the purpose of health professions training, education, or assessment [17]) may provide solutions to increasing training capacity while reducing variability through delivering scalable and asynchronous simulated learning [18-21].

Some evidence suggests VPSs can support health care professionals’ communication skills development [22-27]. For example, typed questions and responses via a virtual patient showed no difference from traditional video scenario-based training for improving suicide risk assessment practices in medical students [23]. VPSs involving unilateral conversation simulators, either with options to select from limited predefined responses to a series of virtual patient questions [25], or learners responding orally to a recorded vignette [22], as well as virtual avatars controlled by a live person [26,27] have also been tested, with varied results. However, the majority of VPS research has used lower fidelity approaches, such as typed text or selective responses [19], or has been undertaken in other contexts such as clinical reasoning.

There remains a paucity of high-quality comparative studies [20,28], particularly studies including financial comparisons [19], to inform the translation of these technologies for developing communication skills into education curricula. Specifically, there has been no research investigating the effectiveness of VPSs using unrestricted 2-way oral conversations. No previous studies have assessed the independent benefit of VPSs for developing communication skills, nor compared them head-to-head with traditional teaching approaches for economic impact, as outlined by previous review articles [19,20].

SimConverse is a web-based artificial intelligence (AI)-driven voice recognition platform designed to develop communication skills in health care professionals. It uses a VPS that interacts with learners through natural language in unrestricted 2-way oral conversations. While there is some evidence of its development and feasibility as an education tool [29], SimConverse has yet to be tested at scale in medical education.

The University of Nottingham (UoN) medical degree has a large undergraduate program with communication skills embedded as a spiral theme throughout its primary care modules, primarily delivered using a conventional approach of actor-based simulation [30]. Thus, providing an opportunity to robustly investigate the implementation of novel technologies for developing communication skills.

Consequently, this study aimed to was to compare an AI-driven voice recognition VPS (SimConverse) with the current standard delivery of communication skills training in undergraduate medical students [30]. We aimed to test differences in self-rated communication skills developed, student satisfaction, and conduct a direct cost comparison of delivering the 2 interventions. We hypothesized that there would be no differences in attainment of self-rated communication skills and student satisfaction between interventions, but that the VPS would have a lower delivery cost.

## Methods

### Design and Setting

A multicenter, open-label randomized crossover study was undertaken during a 1-week primary care attachment, early primary care delivered to 3rd year undergraduate medical students at 2 higher education institutions (UoN and University of Lincoln [UoL]) using the same curriculum.

The students involved were all at the equivalent mid-curricula level of study, having the same knowledge base and having completed the same gateway assessments. Most of their prior training had been classroom-based, other than approximately 2 weeks of clinical training as single or half-day visits spread across 2 years. The attachment on which this study was based was a short bridging attachment to support their transition into full-time clinical training. Students involved were therefore relative novices to communication skills in a clinical context, with this being their first block-based placement in any setting. Due to the comparatively junior stage of students, the focus was on improving relatively basic communication skills, such as history taking, rather than more complex skills such as breaking bad news or dealing with upset or angry patients. As undergraduate students, all are required to use computers and the internet on a regular basis for study and assessment purposes. Therefore, basic computer and internet literacy was assumed for all participants.

Key educational aims of the attachment were to build upon basic skills of communication to communicate effectively with patients in primary care, and to use targeted history taking to aid diagnosis and clinical decision-making as part of a medical consultation.

The study was undertaken using a predetermined protocol and statistical analysis plan and has been reported in line with the CONSORT-EHEALTH V1.6 checklist in the study by Eysenbach and CONSORT-EHEALTH Group [31].

### Participants

Eligible participants were all undergraduate medical students enrolled at UoN or UoL, timetabled to undertake the early primary care attachment. All eligible students were initially included in the study (total cohort recruitment) and were encouraged to take part but were able to opt out. This approach was taken as there were no existing effect sizes to use in a power calculation.

### Randomization

We randomized participants into 2 cohorts (A and B) using a computerized 1:1 random number generator. Participants were subsequently assigned to groups by an administrator who was independent of the study team. The deployment of interventions was unblinded to participants and investigators.

Participants in cohort A were allocated to receive artificial-intelligence communication skills training (AI-CST) first, then actor-based communication skills training (AB-CST) second, with a washout period of 26 hours between the 2 interventions due to timetabling constraints of a total cohort recruitment approach. Cohort B received AB-CST first, then AI-CST second, in parallel to cohort A. Both cohorts completed both interventions.

### Interventions

Both interventions used the same intended learning outcomes (ILOs), namely to: (1) demonstrate the key components of taking a medical history from a patient; (2) listen effectively and respond to a patient’s cues regarding their presenting complaint; (3) begin to integrate biological, psychological, and social factors in assessing a patient’s clinical history; and (4) demonstrate early hypothetico-deductive clinical reasoning in taking a focused medical history of the patient’s presenting complaint.

Although the 2 interventions outlined below have differences, we wanted to compare a new alternative learning methodology (asynchronous virtual learning using a VPS) with the current standard delivery of communication skills training across medical schools (small group actor-based simulation) [30]. We assessed the ability of both “packages of learning” in their individual entireties for developing communication skills. The ILOs across both interventions were identical. Participants were aware that AI-CST was a new technology and, therefore, the “intervention of interest.

#### AI-CST

Participants completed a session of asynchronous unfacilitated AI-CST using the SimConverse platform. The session allowed participants to consult with a virtual patient, receive feedback on their communication, then repeat the consultation up to twice more. In total, participants were provided 3 virtual patients (each with 2 compulsory attempts and a third optional attempt) across the education session. Participants were given a nominal 3 hours in their timetable to complete training. The virtual patients were predeveloped by the research team and designed to be of a comparable level of complexity to those used in the AB-CST intervention. Further details of cases are given in Multimedia Appendix 1.

SimConverse is a purpose-built web-based artificial-intelligence VPS platform designed to develop communication skills in health care professionals [32], accessed via any computer with a microphone and speaker. It allows students to conduct open virtual health care consultations through fully automated high-fidelity audio conversations in real time using voice recognition. The VPS speaks back in real time with text-to-voice. Conversations are anchored using a virtual patient static image. Having completed a consultation, students receive immediate objective grading and personalized feedback on their communication performance using a predeveloped rubric. More detailed product design processes are given in Multimedia Appendix 2.

The SimConverse platform allows faculty to develop their own feedback rubrics or use preexisting ones, based on individual requirements. The SimConverse rubric system and authoring tooling were developed by the company with a multinational group of clinical and educational experts to ensure alignment with best practice marking and feedback. We used a preexisting rubric on the SimConverse platform that was adapted to meet our session ILOs. We tested the rubrics through multiple rounds of initial authoring and previewing in the SimConverse platform as educators, prior to deployment. The exact wording of the rubrics is commercially sensitive data. However, the areas covered in our feedback rubric were the introduction to the consultation, exploration of the history of the presenting complaint, past medical history, drug history, social history, family history, giving a differential diagnosis, giving next steps, and closure of the consultation.

#### AB-CST

Participants also completed traditional AB-CST, representing usual practice as the comparator intervention. AB-CST was delivered as face-to-face small group teaching with 5‐6 participants, a trained actor (the patient), and a trained clinical educator. Each participant completed a different predeveloped medical consultation scenario with a trained actor, lasting 10‐15 minutes. Feedback about communication was provided during the session by the clinical educator, other participants, and the actor.

Overall, participants received a 3-hour session during which they performed 1 consultation each, while observing and discussing feedback for the remaining 5 consultations. Case material and a session outline are given in Multimedia Appendix 1.

### Participant Characteristics

We ascertained details of participant age, gender, widening participation status, and presence of a support plan (covering students with disabilities, long-term medical conditions, including mental health) or specific learning differences, from existing university records, matched to study data using unique participant identifiers. Widening participation refers to a UK initiative designed to increase underrepresented groups in higher education, such as those from low-income backgrounds, first-generation students, ethnic minority students, and students with disabilities. At both UoN and UoL, widening participation students undertake a specific foundation year prior to entering the medical course, which was our marker for their widening participation status.

### Primary Outcome

The primary outcome was the self-reported attainment of communication skills determined using a composite measure of communication assessed through pre-post surveys for both AI-CST and AB-CST.

During trial development, student engagement made clear the need to consider feedback fatigue as we deployed a measurement instrument [33]. A review of literature did not identify any succinct and externally validated tools for measuring the attainment of communication skills to enable comparisons of pre-post intervention scores while simultaneously maximizing participant engagement. Therefore, we developed an in-house survey for measuring self-reported attainment of communication skills, anchored to the Calgary-Cambridge model [34], the communication model taught at UoN and UoL.

The Calgary-Cambridge model is focused on marrying content and process within medical consultations, not excluding either the content of the consultation or the processes of communication at the expense of the other. The framework of its tasks and objectives includes initiating the session, gathering information, physical examination, explanation and planning, and closing the session. Throughout this process, run pillars of providing structure and flow while also building relationships, rapport, and involving the patient (Multimedia Appendix 3). The objectives in the model serve as subheadings to support conceptualization while helping learners organize and apply numerous communication process skills [35]. There are many such skills that might be included, depending on the individual consultation. These might include question style: open to closed, attentive listening, responding to cues, time-framing, summarizing, empathizing, signposting, parking, or many more (Skills for Communicating with Patients [36]). There is evidence of the effectiveness of the Calgary-Cambridge model in assessing communication skills in medical students [37].

**Figure 1.**
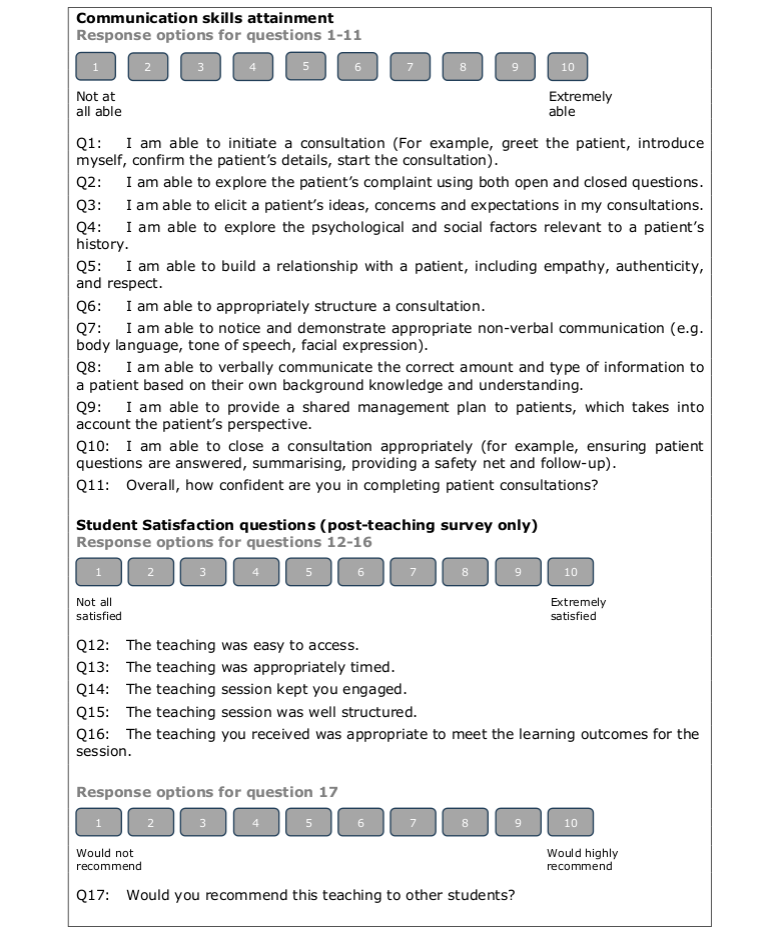
Survey questions for undergraduate medical students to identify self-reported communication skill development pre- and posteducational interventions and satisfaction levels posteducational intervention.

An 11-item survey (Figure 1) was developed using an iterative process by 6 of the research team with extensive medical education experience. How the survey maps to the Calgary-Cambridge model is outlined in Table S1 in Multimedia Appendix 4. Usability and technical functionality were piloted with 5 students, using the method outlined by Arundel [38]. Ten-point linear scales were used to maximize discrimination of pre-post intervention survey scores within individuals [39,40]. Only end points in each survey item were anchored to interpretations, allowing each to be treated as an interval scale, thus allowing parametric statistical hypotheses testing [39,40]. The survey was housed on the Microsoft Forms platform, allowing responses to be downloaded as CSV files for analysis without the need for any manual data entry. No cookies or IP addresses were recorded. Participants were identifiable by username details supporting matching of pre-post surveys and demographic data. Participants were incentivized by being offered entry into a prize draw to win 1 of 3 £100 (US $126.10) shopping vouchers for completing pre- and postsession surveys for both interventions.

For both interventions, participants were asked at the start and end of the session to complete online surveys. Consent was provided by advancing beyond the front screen, which explained the survey with wording preagreed with the ethics committee, including an option to opt out. Participants were provided with detailed study information, including the purpose of the study, matching with institutionally held demographic data, data storage arrangements, and details of the investigator. Preintervention surveys consisted of 2 screens, 1 for consent and the other containing the 11 items. Postintervention surveys contained a third screen with a 6-item satisfaction survey. All survey items were mandatory, although students could opt out at any point by closing the survey. For AB-CST, the surveys were accessed via the provision of QR codes. For AI-CST, links to the appropriate pre-post intervention surveys were built into the SimConverse platform. All participants were sent 2 email reminders to maximize completion of postsession surveys.

Only completed surveys were analyzed. Pre-post changes in aggregated mean scores for both interventions were used as the primary outcome measure and compared across interventions.

### Secondary Outcomes

We measured participant satisfaction with an additional 6 items in postintervention surveys (Figure 1), using 10-point linear scales. Aggregated mean scores for both interventions were used as the composite outcome for satisfaction and compared across interventions.

We also collected data enabling the comparison of steady-state direct costs of delivering the interventions [41], further outlined below.

### Statistical Analyses

Participant characteristics were summarized as numbers (percentages) for categorical data and mean (SD) for continuous data. Demographic characteristics of cohorts A and B were compared to assess the robustness of the randomization process.

#### Primary Analyses

Mean changes in the pre-post intervention ratings were calculated and compared using independent *t* tests (2-tailed). Differences in the aggregated mean scores between the 2 interventions were then compared using independent *t* tests for self-rated communication skill attainment. To avoid excessively restricting the analyses of this within-curriculum randomized study of educational interventions, we included all survey results a priori*,* regardless of whether individual students had completed both pre- and postsession surveys.

We conducted a sensitivity analysis including only data from matched pre-post intervention surveys, while also excluding individuals with first intervention postsession surveys completed after the second intervention had been initiated.

#### Secondary Analyses

To test for the presence of carryover effects, preintervention scores for the second intervention were compared between cohorts A and B. In the presence of carryover, data from the first period before crossover were used to compare treatments in a parallel group design. Differences in aggregated mean scores for satisfaction were also compared between the 2 interventions using independent *t* tests. Analyses were completed using SPSS (version 29; IBM Corp) and *P*<.05 was used to indicate statistical significance.

### Cost Comparison Analyses

The direct steady-state costs of using AI-CST and AB-CST were calculated and compared as the cost per participant taught. For AB-CST, costs included were £200 (US $252.20) per half-day of GP tutor time and £115 (US $145.02) per half-day of actor time. For the full cohort, 54 sessions were delivered. Although exact costs for things such as room hire, administrative time, and electricity costs were difficult to define and therefore not included, 10% of the total staff costs were added as approximate overheads. AI-CST costs were calculated on usage credits (the completion and feedback of 1 attempt at a consultation) and based on 9 allocated credits per student, irrespective of completion rates. SimConverse is a private commercial enterprise and therefore may enter into different licensing agreements with different organizations depending on the number of credits purchased. Therefore, the costs within this study relate to the purchase plan agreed with UoN and costs may vary for other organizations. Further information can be requested directly from the company. For both interventions, nonrecurring setup costs, such as actor and tutor training, scenario development, and technology infrastructure, were not included in the analyses.

### Ethical Considerations

The study was approved by the UoN Faculty of Medicine & Health Sciences Research Ethics Committee (approval number FMHS 43‐1023) on November 20, 2023. Informed consent was obtained from all participants, as previously outlined. All data were collected between January 22 and February 18, 2024. Privacy and confidentiality of participants’ data were maintained at all times. Potential incentives for participants have been outlined under the Primary Outcome section.

## Results

### Response Rates

**Figure 2.**
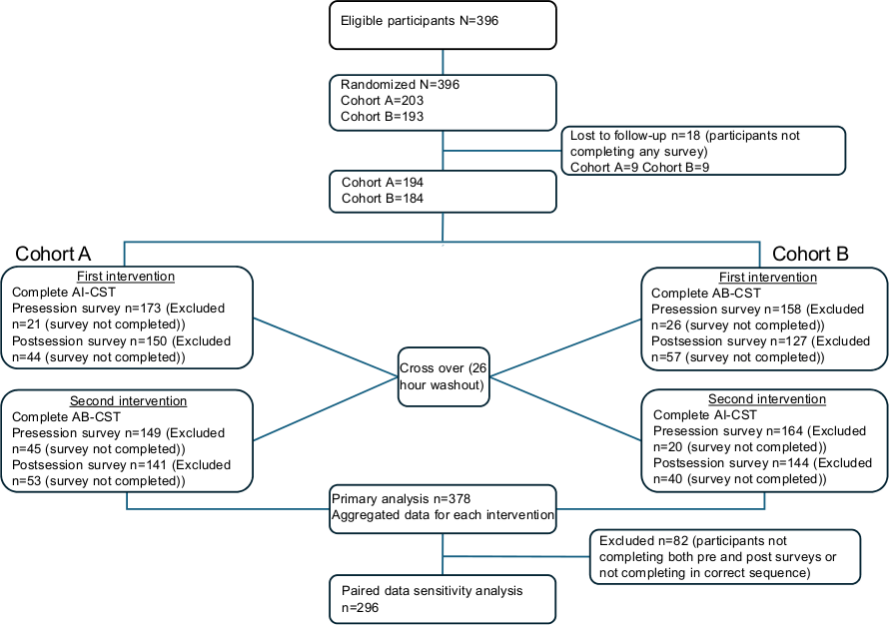
Participant flow and survey responses during the study period for a crossover study examining self-reported communication skill development pre and post both artificial intelligence–based and actor-based communication skills training. AB-CST: actor-based consultation skills training session; AI-CST: artificial intelligence–driven communication skills training session.

In total, 396 (308 from Nottingham and 88 from Lincoln) third-year students were enrolled and eligible to participate at both study sites. Of these, 378 (95%) completed at least 1 survey: 296 (96%) from Nottingham and 82 (93%) from Lincoln. Participant flow and survey responses are provided in Figure 2.

For AI-CST, 85% (337/396) and 74% (294/396) participants completed pre- and postintervention surveys, respectively. For AB-CST, this was 78% (307/396) and 68% (268/396) of participants, respectively. For both interventions, 87% (294/337 for AI-CST and 268/307 for AB-CST) of those completing the preintervention survey also completed the postintervention survey.

There were no differences in the characteristics of participants who completed at least 1 survey between the 2 randomized cohorts (Table 1).

**Table 1.** Characteristics of participants completing at least 1 survey response for self-reported communication skill development pre- or post–artificial intelligence-based or actor-based communication skills training, by randomized group.

Characteristics	Cohort A (n=194)	Cohort B (n=184)
Age (years), mean (SD)	21.69 (1.72)	21.65 (1.44)
Sex, n (%)		
Male	57 (29.4)	55 (29.9)
Female	137 (70.6)	129 (70.1)
Widening participation, n (%)		
Yes	18 (9.3)	21 (11.4)
No	176 (90.7)	163 (88.6)
Support plan, n (%)		
Yes	34 (17.5)	31 (16.8)
No	160 (82.5)	153 (83.2)
Education institution, n (%)		
University of Nottingham	155 (79.9)	141 (76.6)
University of Lincoln	39 (20.1)	43 (23.4)

The time spent on AB-CST had a fixed length of 3 hours due to the timetabled nature of an in-person taught session. AI-CST was asynchronous and unsupervised. It was nominally timetabled for 3 hours, but the mean time participants spent completing AI-CST was 1 hour 53 (SD 45) minutes. Attrition rates for both interventions are given in Multimedia Appendix 5. For AB-CST, 50 participants did not complete the intervention due to absence from teaching, meaning 87% (346/396) completed the intervention as intended. For AI-CST, 69% (272/396) completed the intervention as intended (at least 6 total VPS conversations), with 88% (348/396) completing at least 1 conversation.

### Primary Outcome

Both interventions were found to improve the self-reported attainment of communication skills (Table 2). The pre-post aggregated mean difference scores from AI-CST increased by 1.14 (95% CI 0.97-1.32) points and from AB-CST by 1.50 (95% CI 1.35-1.66) points (both *P*<.001). The comparison of interventions found that the difference in aggregated mean difference scores was 0.36 points lower for AI-CST than AB-CST (95% CI −0.60 to −0.13; *P*=.04).

**Table 2.** Comparison of self-reported communication skills attainment using survey scores pre- and postintervention for both artificial intelligence–based and actor-based communication skills training modalities.

Questions	Participant scores for each question in the communication skills surveys											Primary outcome
	Q1	Q2	Q3	Q4	Q5	Q6	Q7	Q8	Q9	Q10	Q11	
AI-CST^a^ (all responses)												
Mean (SD) baseline (n=337)	8.44 (1.38)	6.87 (1.34)	6.90 (1.45)	6.30 (1.39)	7.35 (1.39)	6.27 (1.60)	7.42 (1.43)	6.37 (1.53)	5.32 (1.84)	6.23 (1.77)	6.25 (1.53)	6.70 (1.16)
Mean (SD) postbaseline (n=294)	9 (1.12)	8.14 (1.21)	8.17 (1.31)	7.63 (1.47)	8.09 (1.50)	7.76 (1.33)	7.60 (1.81)	7.57 (1.52)	6.96 (1.74)	7.74 (1.55)	7.63 (1.24)	7.84 (1.08)
Mean difference (95% CI) from baseline	0.55 (0.34-0.83)	1.27 (1.11-1.59)	1.27 (1.09-1.49)	1.33 (1.14-1.56)	0.74 (0.63-1.18)	1.49 (1.28-1.85)	0.18 (–0.10–0.45)	1.20 (0.96-1.43)	1.64 (1.48-2.17)	1.51 (1.25-1.77)	1.38 (1.16-1.60)	1.14 (0.97-1.32)
*P* value^b^	<.001	<.001	<.001	<.001	<.001	<.001	.18	<.001	<.001	<.001	<.001	<.001
AB-CST^c^ (all responses)												
Mean (SD) baseline (n=307)	8 (1.41)	6.45 (1.23)	6.52 (1.33)	6.14 (1.29)	7.07 (1.30)	5.91 (1.41)	6.93 (1.41)	5.96 (1.46)	4.98 (1.83)	6.02 (1.65)	5.85 (1.37)	6.35 (1.01)
Mean (SD) postbaseline (n=268)	8.88 (1.04)	8.03 (1.05)	8.09 (1.10)	7.66 (1.28)	8.31 (1.14)	7.70 (1.19)	8.15 (1.18)	7.54 (1.26)	6.79 (1.62)	7.63 (1.47)	7.63 (1.04)	7.86 (0.93)
Mean difference (95% CI) from baseline	0.88 (0.65-1.08)	1.58 (1.39-1.78)	1.57 (1.28-1.73)	1.52 (1.25-1.72)	1.24 (0.95-1.42)	1.80 (1.57-2.02)	1.20 (0.98-1.42)	1.62 (1.39-1.84)	1.83 (1.53-2.13)	1.64 (1.37-1.90)	1.78 (1.56-1.99)	1.50 (1.35-1.66)
*P* value^d^	<.001	<.001	<.001	<.001	<.001	<.001	<.001	<.001	<.001	<.001	<.001	<.001
Comparison of AI-CST and AB-CST mean differences												
Mean difference (95% CI) AI-CST	0.55 (0.34-0.83)	1.27 (1.11-1.59)	1.27 (1.09-1.49)	1.33 (1.14-1.56)	0.74 (0.63-1.18)	1.49 (1.28-1.85)	0.18 (–0.10–0.45)	1.20 (0.96-1.43)	1.64 (1.48-2.17)	1.51 (1.25-1.77)	1.38 (1.16-1.60)	1.14 (0.97-1.32)
Mean difference (95% CI) AB-CST	0.88 (0.65-1.08)	1.58 (1.39-1.78)	1.57 (1.28-1.73)	1.52 (1.25-1.72)	1.24 (0.95-1.42)	1.80 (1.57-2.02)	1.20 (0.98-1.42)	1.62 (1.39-1.84)	1.83 (1.53-2.13)	1.64 (1.37-1.90)	1.78 (1.56-1.99)	1.50 (1.35-1.66)
Difference (95% CI) in differences	−0.33 (−0.61 to −0.05)	−0.31 (−0.58 to 0.03)	−0.30 (−0.58 to 0.01)	−0.16 (−0.49 to 0.12)	−0.42 (−0.79 to −0.19)	−0.31 (−0.61 to 0.01)	−1.02 (−1.38 to −0.71)	−0.42 (−0.71 to 0.07)	−0.19 (−0.23 to 0.56)	−0.13 (−0.26 to 0.46)	−0.40 (−0.68 to 0.10)	−0.36 (−0.60 to −0.13)
*P* value^d^	.02	.01	.002	.04	<.001	.06	<.001	0.001	0.09	0.98	<.001	.04

a AI-CST: artificial-intelligence communication skills training.

b Paired *t* test (2-tailed).

c AB-CST: actor-based communication skills training.

d Independent *t* test (2-tailed).

Sensitivity analyses results (Multimedia Appendix 6) using only matched pre-post surveys and excluding participants who completed the first postintervention survey after the second intervention initiation found no change to the primary findings.

### Secondary Outcomes

#### Student Satisfaction

Satisfaction scores were high for both interventions (Table 3) but significantly lower for AI-CST than AB-CST, across all questions. Aggregated mean scores were 8.09 (SD 1.51) and 9.21 (SD 0.92) for AI-CST and AB-CST, respectively (aggregated mean 1.13 points lower, 95% CI −1.33 to −0.92 for AI-CST than AB-CST; *P*<.001).

**Table 3.** Student satisfaction scores and comparison for artificial intelligence-based and actor-based communication skills training modalities.

Questions	Participant scores for each question in the student satisfaction surveys						Aggregated mean (95% CI)
Q12 Easy to access	Q13 Appropriately timed	Q14 Kept you engaged	Q15 Well structured	Q16 Met learning outcomes	Q17 Would recommend to others
AI-CST^a^ mean (SD) score	8.76 (1.34)	8.08 (1.78)	7.45 (2.17)	7.93 (1.76)	8.26 (1.57)	8.03 (1.88)	8.09 (1.51)
AB-CST^b^ mean (SD) score	9.26 (1.02)	9.03 (1.28)	9.11 (1.12)	9.26 (1.00)	9.28 (0.96)	9.34 (0.95)	9.21 (0.92)
Mean difference (95% CI)	−0.5 (−0.70 to −0.13)	−0.95 (−1.20 to −0.69)	−1.66 (−1.94 to −1.38)	−1.33 (−1.57 to −1.10)	−1.02 (−1.23 to −0.81)	−1.31 (−1.55 to −1.06)	−1.13 (−1.33 to −0.92)
*P* value^c^	<.001	<.001	<.001	<.001	<.001	<.001	<.001

a AI-CST: artificial-intelligence communication skills training.

b AB-CST: actor-based communication skills training.

c Independent *t* test (2-tailed).

#### Direct Costs

The steady-state direct cost of delivering AI-CST (£33.48; US $42.22 per student) was substantially lower than for AB-CST (£61.75; US $77.87 per student).

### Carryover Effect Between Interventions

Cohort analyses suggested no differential carryover effect between the interventions (Multimedia Appendix 7). The comparison of preintervention mean scores within both cohorts A and B, prior to the second intervention, found no significant difference between groups (Cohort A: pre-second intervention mean score 6.26, SD 0.86; Cohort B: pre-second intervention mean score 6.62, SD 0.83; mean difference 0.35, 95% CI −1.11 to 0.40; *P*=.34).

## Discussion

### Principal Findings

This is the first randomized study comparing the self-reported effectiveness and direct costs of a VPS using unrestricted 2-way oral conversations with traditional AB-CST for attaining communication skills in undergraduate medical students. Although both interventions showed improvements in self-rated consultation skills and confidence among students, the magnitude of improvement was lower for AI-CST than AB-CST (mean improvement 1.14, 95% CI 0.97-1.32 for AI-CST vs 1.50, 95% CI 1.35-1.66 for AB-CST, respectively), just reaching statistical significance (*P*=.04). Student satisfaction was lower for AI-CST than AB-CST (aggregated mean score 1.13 points lower, 95% CI –1.33 to –0.92; *P*<.001), although satisfaction for both interventions was generally high. AI-CST was approximately half the cost of AB-CST.

### Findings in Context

Simulation-based learning is effective in clinical training [42-45], while allowing for skills development without compromising patient safety [46]. The slightly larger benefit and higher satisfaction we demonstrated from AB-CST may link to medical student perspectives, that patient interactions supporting development of interpersonal skills and understanding of patient-centered care are vital to them [47]. While AI-CST does include elements of this, we surmise that the richness of opportunities and feedback linked to interpersonal skills and understanding of patient-centered care would be richer from AB-CST than from VPS interactions.

Previous studies have examined VPSs for developing clinical reasoning in medical students. These were small and investigated text-based interfaces rather than verbal communication [48-51]. While they found improvements in student knowledge of clinical reasoning, they did not investigate communication skills development.

A series of small US-based randomized trials has investigated virtual patients to train undergraduate medical students in communication skills. However, most used scripted textual question responses and not higher fidelity voice recognition or 2-way verbal conversations as encountered in live clinical environments [22,23,52].

Foster et al [23] showed that a single online-based virtual patient simulation was as effective as video-based teaching for training 67 medical students in conducting a suicide risk assessment. Similar to our findings, the VPS scored lower in student satisfaction than the video-based teaching. Another trial of 99 medical students using video-based simulations with typed questions from a predefined list showed a virtual patient to be as effective at developing communication skills in medical students as traditional learning. However, the comparison group was ‘education as normal’, with no distinct comparison intervention [52]. In contrast, a trial of 84 medical students showed that a VPS with scripted responses to limited verbal student questions was less effective at developing nonverbal communication skills than an actor-based simulation with the same scripted responses [22].

One study investigated an AI-developed voice-activated VPS using natural language for delivering feedback about medical student consultations. The VPS was as effective as humans at marking and providing feedback on performance, using a specified marking rubric [24], but its ability to improve communication skills was not examined.

The largest study by Kron et al [25], of 421 medical students, found that a VPS was more effective at developing communication skills than a computer-based training package using text, image, and video. Of note, the VPS, although using spoken language, only allowed students to select from 3 predefined responses during conversations, limiting the fidelity of the VPS to mimic live clinical patient encounters. In addition, the comparator in this study was atypical of usual practice for developing communication skills and did not allow students to practice the skills they had learned with any sort of patient interaction, human or otherwise. This may explain the contrast with the results shown in our study.

One of the challenges for technology-enhanced learning in communication skills training is its ability to support the development of interpersonal skills and nonverbal communication [53,54]. Guetterman et al [55], in a further analysis of data from the Kron study outlined above, reported positive impacts from the VPS on nonverbal cues demonstrated by students. However, the technology was limited to detecting simple movements, such as nodding, smiling, and eyebrow raising, and may overlook other nonverbal cues and expressions of empathy.

Other studies using VPSs with scripted textual responses have indicated their potential benefits for training students in shared decision-making [56], improving learner confidence in patient interactions [57], improving objective structured clinical examination performance [58], and that they evoke similar emotional responses when breaking bad news to real patient interactions [59].

### Strengths and Limitations

To our knowledge, this is the first global within-curricula study to investigate the self-reported effectiveness of a VPS using unrestricted 2-way verbal conversation for developing communication skills in undergraduate medical students. A strength was the randomized crossover design and pre-post assessment of outcomes in the study population, enabling the control of confounders and differences in scenario complexity of interventions. The large multicenter design and high response rates increase the generalizability of findings to undergraduate medical education. Uniquely, we calculated the attributable intervention costs, providing greater insight to education providers about the implementation of such interventions into routine practice.

We did not complete an a priori sample size calculation as there were no outcome data for effectiveness or effect size across interventions. However, we conducted a post hoc power calculation from our results, suggesting our study had 99.8% power to detect differences between the interventions at the 5% significance level. While there was a short wash-out period in the crossover design of this study, our secondary analyses were reassuring, suggesting there was no carryover effect, and our primary analyses were valid.

There were some limitations. The deployment of interventions was unblinded to participants and investigators, with outcomes self-reported, thus introducing the potential for responder and verification biases within findings. Objective outcomes, such as communication skills attainment from summative assessments, would have strengthened the findings. It is plausible that an element of the Dunning-Kruger effect may have impacted our findings, with low ability students overestimating their competence, and more able students underestimating theirs [60]. This would have impacted our results toward the null, resulting in us underestimating the actual difference in effect size between the 2 interventions. The pre-post survey design of our study should control for within-individual biases and therefore adjust for this. However, within medical education studies that use self-reporting instruments, it may be difficult to completely remove this effect.

We were able to report adherence rates to both interventions. However, we were not able to establish this at a participant level and therefore could not examine an association between this and learner confidence. This would be a relevant consideration for future research.

The single-session exposure to interventions was short. Therefore, the incremental impact and sustained improvements in communication skills from longer exposures could not be determined. The setting of this trial was in a short primary care-based attachment lasting only 1 week. It has provided evidence that supports a “proof-of-concept,” although with a limited outcome measure. Our findings support the next step to embed this technology into curricula over a sustained period, thus enabling assessment against summative education outcomes. However, this would paradoxically introduce other methodological challenges, such as controlling for the effects of learning and development of communication skills that may arise from other curricula opportunities beyond the use of such technology.

The statistical precision could have been improved by using only paired survey responses. However, we wanted to use all available data in our primary analyses to maximize study power and reflect the testing of a new educational intervention in a live ‘real world’ learning environment. Our sensitivity analyses included only the paired responses and showed no change from the primary findings. This study was conducted at a single time point during medical training, and we would caution against the extrapolation of findings to students at different stages of medical training.

### Conclusions

AI-CST using a voice recognition–based VPS was effective in improving self-reported communication skills in undergraduate medical students. However, it was not as effective as AB-CST. Student satisfaction for AB-CST was significantly better than AI-CST. The cost of AI-CST was roughly half that of AB-CST.

AI-CST may therefore provide a cost-effective solution to building capacity for teaching communication in health care professionals. Further research is needed to determine how the sustained improvement of communication skills can be developed using AI-CST, and the testing of such technology with students at other stages of medical training and in wider professional groups.

## Supplementary material

10.2196/71667 Multimedia Appendix 1 Case Material for artificial intelligence–based and actor-based communication skills training interventions.

10.2196/71667 Multimedia Appendix 2 SimConverse (artificial intelligence–based communication skills training) product design information.

10.2196/71667 Multimedia Appendix 3 The Calgary Cambridge model (adapted from Kurtz et al, 2003).

10.2196/71667 Multimedia Appendix 4 Mapping of survey instrument for measuring self-reported communication skill development pre- and posteducational interventions in undergraduate medical students to Calgary-Cambridge model.

10.2196/71667 Multimedia Appendix 5 Attrition rates of participants during the study period for both AI-based and actor-based communication skills training modalities.

10.2196/71667 Multimedia Appendix 6 Sensitivity analysis of comparison of self-reported communication skills attainment using survey scores pre and postintervention for both artificial intelligence-based and actor-based communication skills training modalities, including only data from matched pre-post intervention surveys, and excluding surveys completed out of sequence.

10.2196/71667 Multimedia Appendix 7 Within cohort analyses for the carryover effect between artificial intelligence-based and actor-based communication skills training interventions in the crossover study design.

10.2196/71667 Checklist 1 CONSORT eHEALTH checklist.

## Abbreviations

AB-CST: actor-based consultation skills training
AI: artificial intelligence
AI-CST: artificial intelligence-based communication skills training
ILO: intended learning outcome
UoL: University of Lincoln
UoN: University of Nottingham
VPS: virtual patient simulator
