# Supplementary material for: Web-Based AI-Driven Virtual Patient Simulator Versus Actor-Based Simulation for Teaching Consultation Skills: Multicenter Randomized Crossover Study
Source: JMIR Form Res. 2025 Nov 20;9:e71667. doi: 10.2196/71667 (PMC12634008; doi:10.2196/71667)
Supplement: Multimedia Appendix 1 [file formative-v9-e71667-s001.docx]

**Key Instructions and Case Material for AI-CST session**

The exact character prompts within the SimConverse platform that instruct the VPS of the character details and how to respond are commercially sensitive, privately owned intellectual property. For further information, please contact SimConverse directly.

Individual cases (characters) can be utilised from pre-existing ones in the platform, or be developed from scratch by faculty. SimConverse provides training in character development within the platform, once a licensing agreement is entered into.

A summary of the cases and steps involved in the SimConverse session delivered as part of this study are given below.

| **Case** | **Clinical details** | **Likely diagnosis** | **Steps** |
| --- | --- | --- | --- |
|  |  |  | - Initial tutorial on how to use the platform (one-off) |
| Case 1 | 47 year old male presenting to Emergency Department with abdominal pain and vomiting | Alcohol induced pancreatitis | - Pre-brief, including set up for psychological safety information and brief background information on patient - Conversation with patient - Review of conversation transcript and feedback rubric (includes pass/fail criteria for each element of rubric) - Written reflection - Conversation with patient (attempt 2) - Review of conversation 2 transcript and feedback rubric (includes pass/fail criteria for each element of rubric) - **Optional** conversation with patient (attempt 3) - Review of conversation 3 transcript and feedback rubric (includes pass/fail criteria for each element of rubric) - Final written reflection |
| Case 2 | 87 year old male presenting to primary care with fatigue and worsening leg swelling | Congestive cardiac failure | - Pre-brief, including set up for psychological safety information and brief background information on patient - Conversation with patient - Review of conversation transcript and feedback rubric (includes pass/fail criteria for each element of rubric) - Written reflection - Conversation with patient (attempt 2) - Review of conversation 2 transcript and feedback rubric (includes pass/fail criteria for each element of rubric) - **Optional** conversation with patient (attempt 3) - Review of conversation 3 transcript and feedback rubric (includes pass/fail criteria for each element of rubric) - Final written reflection |
| Case 3 | 68 year old male presenting to primary care with worsening cough and shortness of breath over the last 4 days | Community acquired pneumonia | - Pre-brief, including set up for psychological safety information and brief background information on patient - Conversation with patient - Review of conversation transcript and feedback rubric (includes pass/fail criteria for each element of rubric) - Written reflection - Conversation with patient (attempt 2) - Review of conversation 2 transcript and feedback rubric (includes pass/fail criteria for each element of rubric) - **Optional** conversation with patient (attempt 3) - Review of conversation 3 transcript and feedback rubric (includes pass/fail criteria for each element of rubric) - Final written reflection |

**Key Instructions and Case Material for AB-CST session**

This teaching material is part of the standard teaching curriculum at University of Nottingham.

| **Step by step guide for sessions**  These steps have been written in order to assist the tutor to facilitate as smoothly as possible.   - **Share the ‘mini-surgery’** on the screen. - Explain to students that these are the patients you will work through in the session. The students have had access to this in advance to prepare for with the case title and “information for doctors”. - If you use **‘time out’** as an option for students explain the concept/how they can signal this. - **Everyone will get the chance to consult once.** Ask for a volunteer/nominate *A Student* to go first, but everyone will get a chance. - Get someone to read out **the first case –** you could put up the slide, or get one of the students to read from [University Online Learning Platform]. - Ask the consulting student if there **are any elements in this information they wish to clarify** - Ask the consulting student what **particular areas of their consultation they wish for others to watch out for/feedback on**. - Give **clear instructions** to the students giving **feedback** on what you would like them to comment on based on the learner’s agenda and the case. See above for further guidance on ALOBA/feedback depth. - **Nominate a timekeeper** (you may decide it is best to be you). Then ask the consulting student whether they want a verbal warning on timing during the consultation (usually at 5 minutes and 9 minutes, if so). They may decide they would prefer not to have this. - There is always a balance between allowing a student to complete and close down a consultation, even if a little over-run, with keeping to time and ensuring time to complete the 6 cases. Perhaps allow up to around 12 minutes from the start of the case if necessary, but draw the student to a halt if they are continuing beyond this. You may choose to do differently. - **Having finished, ask the student to briefly present the case – this is a very useful skill for students to practice at this stage** - This leaves the rest of the 25 minute period for that case (likely 10-15 mins) for feedback/discussion - **Elicit feedback** from students as you have outlined with their specific areas to monitor. **Don’t forget the patient**, who will be an excellent source of feedback! See below for more suggestions on feedback. - Now move onto the **next case**. **Share the case** notes and repeat. |
| --- |

A summary of key points and the information given to actors and clinical tutors for each case is summarised below. Exact details of character prompts to actors can be provided on specific request from the authors:

| **Case** | **Clinical details** | **Likely diagnosis** | **Further information provided to actor** |
| --- | --- | --- | --- |
| Case 1 | 58 year old patient (male or female patient, depending on gender of actor) presenting with troublesome cough for 6 months | Chronic Obstructive Airway Disease | - Reason for attendance – “This cough just won’t go away” - Additional clinical details - Details to only give if specifically asked (relating to red flag symptoms) - Past medical history - Drug history - Social history - Family history - Patient ideas, concerns and expectations |
| Case 2 | 47 year old patient (male or female patient, depending on gender of actor) with headaches increasing in frequency | Migraines | - Reason for attendance – “My headaches are getting more frequent” - Additional clinical details - Details to only give if specifically asked (relating to red flag symptoms) - Past medical history - Drug history - Social history - Family history - Patient ideas, concerns and expectations |
| Case 3 | 64 year old patient (male or female patient, depending on gender of actor) with 3 months of intermittent chest pain symptoms | Angina pectoris | - Reason for attendance – “This pain in my chest is starting to bother me” - Additional clinical details - Details to only give if specifically asked (relating to red flag symptoms) - Past medical history - Drug history - Social history - Family history - Patient ideas, concerns and expectations |
| Case 4 | 49 year old patient (male or female patient, depending on gender of actor) with 6 months of intermittent abdominal pain worsening in the last 1 week | Biliary colic | - Reason for attendance – “My tummy pain is getting worse” - Additional clinical details - Details to only give if specifically asked (relating to red flag symptoms) - Past medical history - Drug history - Social history - Family history - Patient ideas, concerns and expectations |
| Case 5 | 55 year old patient (male or female patient, depending on gender of actor) with 6 months of increasing knee pain | Osteoarthritis of the knee | - Reason for attendance – “My knee is really getting me down” - Additional clinical details - Details to only give if specifically asked (relating to red flag symptoms) - Past medical history - Drug history - Social history - Family history - Patient ideas, concerns and expectations |
| Case 6 | 52 year old patient (male or female patient, depending on gender of actor) with 3 weeks of persistent diarrhoea symptoms | Infective diarrhoea | - Reason for attendance – “I’ve had the runs for about 3 weeks and it’s not getting any better” - Additional clinical details - Details to only give if specifically asked (relating to red flag symptoms) - Past medical history - Drug history - Social history - Family history - Patient ideas, concerns and expectations |
