# Supplementary material for: Web-Based AI-Driven Virtual Patient Simulator Versus Actor-Based Simulation for Teaching Consultation Skills: Multicenter Randomized Crossover Study
Source: JMIR Form Res. 2025 Nov 20;9:e71667. doi: 10.2196/71667 (PMC12634008; doi:10.2196/71667)
Supplement: Multimedia Appendix 2 [file formative-v9-e71667-s002.docx]

The study authors have had no role in the development of the SimConverse technology and do not have any financial or other interests in SimConverse. The SimConverse team has in part, provided the detail below, as the study team would otherwise not have had access to all this information, including some technical details.

SimConverse has been in production since August 2020 and is a software-as-a-service (SaaS) product available to organisations for purchase.

The SimConverse platform functions by orchestrating multiple proprietary AI models. Each model performs a single modality transform (e.g. speech->text, text->text, text->speech etc.). All models utilise an autoregressively sampled (i.e. generative) Transformer decoder (2017, Vaswani et al) and a modality-specific encoder. This separation of modalities was performed in contrast to fused multi-modal approaches in order to provide a higher degree of interpretability & control. Generative sampling was selected in order to provide a robust experience for learners, in contrast to classification-based NLP, which requires hand coding every individual VSP encounter. All AI models were trained on a proprietary data format designed to significantly minimise the incidence of clinically relevant ‘hallucinations’ in the trained models compared to publicly available foundation models such as ChatGPT.

SimConverse is a web application that does not require any downloads. It can be easily accessed via any web browser, from any electronic device with a microphone and speaker, provided the device meets the minimum requirements to run a basic web browser.

**Costs**

As SimConverse is a private commercial enterprise there are variable licensing costs according to purchasing structures with individual organisations. Therefore, the costs within this study relate to the purchase plan agreed with University of Nottingham and costs may vary for other organisations. Further information can be requested directly from the company.

**Feedback rubrics**

This detail is described within the Methods section.

**Case Material**

The exact character prompts within the SimConverse platform that instruct the VPS of the character details and how to respond are commercially sensitive, privately owned intellectual property. For further information, please contact SimConverse directly.

Individual cases (characters) can be utilised from pre-existing ones in the platform, or be developed from scratch by faculty. SimConverse provides training in character development within the platform, once a licensing agreement is entered into.

A summary of the cases and steps involved in the SimConverse session delivered as part of this study are given in Supplementary File S1.
