# Supplementary material for: Web-Based AI-Driven Virtual Patient Simulator Versus Actor-Based Simulation for Teaching Consultation Skills: Multicenter Randomized Crossover Study
Source: JMIR Form Res. 2025 Nov 20;9:e71667. doi: 10.2196/71667 (PMC12634008; doi:10.2196/71667)
Supplement: Multimedia Appendix 3 [file formative-v9-e71667-s003.docx]

**Figure S1.**


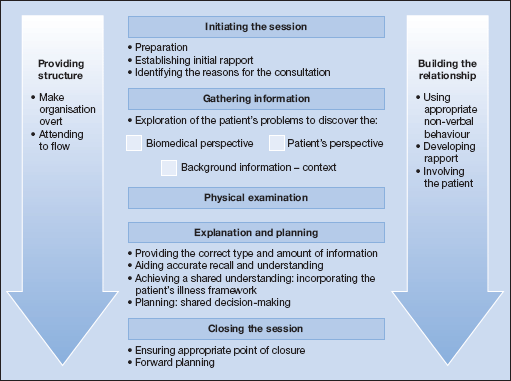


**Reference**

1. Kurtz S, Silverman J, Benson J, Draper J. Marrying Content and Process in Clinical Method Teaching: Enhancing the Calgary–Cambridge Guides. Academic Medicine. 2003;78(8).
