# Supplementary material for: Web-Based AI-Driven Virtual Patient Simulator Versus Actor-Based Simulation for Teaching Consultation Skills: Multicenter Randomized Crossover Study
Source: JMIR Form Res. 2025 Nov 20;9:e71667. doi: 10.2196/71667 (PMC12634008; doi:10.2196/71667)
Supplement: Multimedia Appendix 4 [file formative-v9-e71667-s004.docx]

**Table S1.**

| **Calgary-Cambridge Model Headings** | **Survey question:** | **Survey response options** | | | | | | | | | |
| --- | --- | --- | --- | --- | --- | --- | --- | --- | --- | --- | --- |
|  | **Please rate your overall ability to do the following things when undertaking consultations with patients:** | Not at all able | | |  |  |  |  | Extremely able | | |
|  |  | 1 | 2 | 3 | 4 | 5 | 6 | 7 | 8 | 9 | 10 |
| Initiating the session | I am able to initiate a consultation (For example, greet the patient, introduce myself, confirm the patient’s details, start the consultation) |  |  |  |  |  |  |  |  |  |  |
| Gathering information – biomedical perspective | I am able to explore the patient’s problem using both open and closed questions |  |  |  |  |  |  |  |  |  |  |
| Gathering information – patient’s perspective | I am able to elicit a patient’s ideas, concerns and expectations in my consultations |  |  |  |  |  |  |  |  |  |  |
| Gathering information – background information (context) | I am able to explore the psychological and social factors relevant to a patient’s history |  |  |  |  |  |  |  |  |  |  |
| Building the relationship | I am able to build a relationship with a patient, including empathy, authenticity and respect |  |  |  |  |  |  |  |  |  |  |
| Providing structure | I am able to appropriately structure a consultation |  |  |  |  |  |  |  |  |  |  |
| Building the relationship | I am able to pick up and demonstrate appropriate non-verbal communication (e.g. body language, tone of speech, facial expression) |  |  |  |  |  |  |  |  |  |  |
| Explanation and planning | I am able to provide the correct amount and type of information for an individual patient, based on their own background knowledge |  |  |  |  |  |  |  |  |  |  |
| Explanation and planning | I am able to provide a shared management plan to patients, which takes into account the patient’s perspective |  |  |  |  |  |  |  |  |  |  |
| Closing the session | I am able to close a consultation appropriately (for example, ensuring patient questions are answered, summarising, providing a safety net and follow-up) |  |  |  |  |  |  |  |  |  |  |
|  |  | Not at all confident | | |  |  |  |  | Extremely confident | | |
|  |  | 1 | 2 | 3 | 4 | 5 | 6 | 7 | 8 | 9 | 10 |
| Overall | Overall, how confident are you in completing patient consultations? |  |  |  |  |  |  |  |  |  |  |
