# Supplementary material for: Web-Based AI-Driven Virtual Patient Simulator Versus Actor-Based Simulation for Teaching Consultation Skills: Multicenter Randomized Crossover Study
Source: JMIR Form Res. 2025 Nov 20;9:e71667. doi: 10.2196/71667 (PMC12634008; doi:10.2196/71667)
Supplement: Multimedia Appendix 5 [file formative-v9-e71667-s005.docx]

**Figure S1.**

Abbreviations: ABCST, Actor-based communication skills training; AICST, Artificial intelligence-based communication skills training

Dotted line indicates consultations that were optional to participants and therefore not part of the standard package of learning for AI-CST
