# Supplementary material for: Web-Based AI-Driven Virtual Patient Simulator Versus Actor-Based Simulation for Teaching Consultation Skills: Multicenter Randomized Crossover Study
Source: JMIR Form Res. 2025 Nov 20;9:e71667. doi: 10.2196/71667 (PMC12634008; doi:10.2196/71667)
Supplement: Multimedia Appendix 6 [file formative-v9-e71667-s006.docx]

**Table S1.**

| **Question** | **Mean participant scores for each question in the communication skills surveys** | | | | | | | | | | | **Primary outcome:**  **Aggregated mean score**  **(95%CI)** |
| --- | --- | --- | --- | --- | --- | --- | --- | --- | --- | --- | --- | --- |
|  | **Q1** | **Q2** | **Q3** | **Q4** | **Q5** | **Q6** | **Q7** | **Q8** | **Q9** | **Q10** | **Q11** |  |
| **AI-CST^a^ (222 responses, Group A n=120, Group B n= 102)** | | | | | | | | | | | | |
| Mean prebaseline (SD) | 8.40  (1.39) | 6.89  1.28) | 6.92  (1.43) | 6.30  (1.40) | 7.42  (1.43) | 6.34  (1.52) | 7.43  (1.43) | 6.38  (1.51) | 5.39  (1.82) | 6.19  (1.73) | 6.29  (1.49) | **6.72**  **(0.79)** |
| Mean postbaseline (SD) | 9.00  (1.10) | 8.16  (1.22) | 8.12  (1.33) | 7.60  (1.49) | 8.05  (1.50) | 7.82  (1.32) | 7.51  (1.82) | 7.52  (1.54) | 6.92  (1.80) | 7.72  (1.50) | 7.60  (1.29) | **7.82**  **(0.54)** |
| Mean difference from baseline (95% CI) | 0.6 | 1.27 | 1.20 | 1.30 | 0.63 | 1.48 | 0.08 | 1.14 | 1.53 | 1.53 | 1.31 | **1.10 (0.78 to 1.41)** |
| *P* value^b^ | **<.001** | **<.001** | **<.001** | **<.001** | **<.001** | **<.001** | **<.001** | **<.001** | **<.001** | **<.001** | **<.001** | **<.001** |
| **AB-CST^c^ (226 responses, Group A n=119, Group B n=107)** | | | | | | | | | | | | |
| Mean prebaseline (SD) | 7.99  (1.37) | 6.42  (1.13) | 6.51  (1.24) | 6.08  (1.22) | 7.03  (1.29) | 5.95  (1.32) | 6.93  (1.36) | 5.93  (1.36) | 4.94  (1.81) | 6.02  (1.56) | 5.85  (1.31) | **6.33**  **(0.79)** |
| Mean postbaseline (SD) | 8.86  (1.05) | 8.00  (1.01) | 8.07  (1.09) | 7.64  (1.18) | 8.23  (1.17) | 7.66  (1.16) | 8.08  (1.29) | 7.51  (1.29) | 6.72  (1.63) | 7.55  (1.48) | 7.60  (1.04) | **7.81**  **(0.54)** |
| Mean difference from baseline (95% CI) | 0.870 | 1.580 | 1.560 | 1.560 | 1.200 | 1.710 | 1.150 | 1.580 | 1.780 | 1.530 | 1.750 | **1.48**  **(1.29 to 1.67)** |
| *P* value^b^ | **<.001** | **<.001** | **<.001** | **<.001** | **<.001** | **<.001** | **<.001** | **<.001** | **<.001** | **<.001** | **<.001** | **<.001** |
| **Comparison of AI-CST & AB-CST mean differences** | | | | | | | | | | | | |
| Mean difference AI-CST | 0.60 | 1.27 | 1.20 | 1.30 | 0.63 | 1.48 | 0.08 | 1.14 | 1.53 | 1.53 | 1.31 | **1.10** |
| Mean difference AB-CST | 0.87 | 1.58 | 1.56 | 1.56 | 1.20 | 1.71 | 1.15 | 1.58 | 1.78 | 1.53 | 1.75 | **1.48** |
| Difference in differences | -0.27  (-0.49 to  -0.05) | -0.31  (-0.55 to  -0.10) | -0.36  (-0.59 to  -0.10) | -0.26  (-0.54 to  -0.01) | -0.57  (-0.83 to  -0.29) | -0.23  (-0.51 to 0.02) | -1.07  (-1.38 to  -0.78) | -0.44 (-0.71 to  -0.21) | -0.25  (-0.60 to 0.05) | 0.00  (-0.33 to 0.29) | -0.44  (-0.67 to  -0.22) | **-0.38**  **(-0.56 to -0.20)** |
| *P* value^d^ |  |  |  |  |  |  |  |  |  |  |  | **0.03** |

^a^AI-CST: artificial-intelligence communication skills training

^b^Paired t-test

^c^AB-CST: actor-based communication skills training

^d^Independent t-test
