# Supplementary material for: Web-Based AI-Driven Virtual Patient Simulator Versus Actor-Based Simulation for Teaching Consultation Skills: Multicenter Randomized Crossover Study
Source: JMIR Form Res. 2025 Nov 20;9:e71667. doi: 10.2196/71667 (PMC12634008; doi:10.2196/71667)
Supplement: Multimedia Appendix 7 [file formative-v9-e71667-s007.docx]

**Figure S1.**

Group A = Artificial Intelligence-based communication skills training (AI-CST) first intervention and actor-based communication skills training (AB-CST) second intervention

Group B = Actor-based communication skills training (AB-CST) first intervention and artificial Intelligence-based communication skills training (AI-CST) second intervention
